# Early diagnostic value of intima-media thickness and D-dimer levels for vascular dementia

**DOI:** 10.1097/MD.0000000000034149

**Published:** 2023-06-23

**Authors:** Haiping Xia, Biao Jin, Cen Su, Kangren Zhao, Rui Ma

**Affiliations:** a Zhenjiang 4th People’s Hospital Affiliated to Jiangsu University, Jiangsu, China; b Department of Anatomy, Medical School, Jiangsu University, Jiangsu, China.

**Keywords:** cognitive decline, intima-media thickness, vascular dementia

## Abstract

To evaluate the diagnostic value of combination of D-dimer and Doppler Intima-Media Thickness (IMT) based on clinical data for vascular dementia (VaD). In this study, 100 cases of initial VaD were diagnosed by Zhenjiang Fourth People Hospital as VaD group, and 100 healthy were engaged as control group, medical history and test results were collected for evaluation. IMT and D-dimer were highly correlated with the onset of VaD. The sensitivity of combination diagnosis for early VaD: 90.2%, the specificity: 87.9%, the area under the curve (AUC) is 0.872, which were superior to IMT or D-dimer alone. D-dimer and IMT are significantly increased in the initial onset of cerebrovascular diseases, and combined detection of them is conducive to early diagnosis and evaluation of the disease. Given the limitations of D-dimer and IMT, combined detection is more conducive to early diagnosis and prognosis, and can be used as screening and routine examination items, even evaluate the severity of cognitive decline.

## 1. Introduction

The diagnosis of vascular dementia (VaD) only depends on symptoms and images nowadays, so it is necessary and challenge to find recognized and specific indexes for early and differential diagnosis.^[1]^ The causes and mechanisms of VaD are far from knowing, which brings difficulties for researchers and doctors for VaD.^[1,2]^

D-dimer is not only a hemocoagulatory factor but also a inflammatory factors, plays an important role in VaD development, at the same time, Intima-media thickness (IMT) is a direct indicator of pathological imaging of cerebrovascular diseases, which can not only reflect the occurrence and development of atherosclerosis but also can predict cognitive decline in time.^[3]^ Because of the limitations of single test,^[3]^ we used clinical data to determine the value of the combination of D-dimer and IMT in the evaluation of VaD, providing evidence for diagnosis, treatment and prognosis. At the same time, D-dimer, unlike other biomarkers,^[4,5]^ which is a molecule secreted by inflammatory cells that can promote the development of inflammation.

## 2. Subjects and methods of study

### 2.1. Basic information in this study

**Figure 1.**
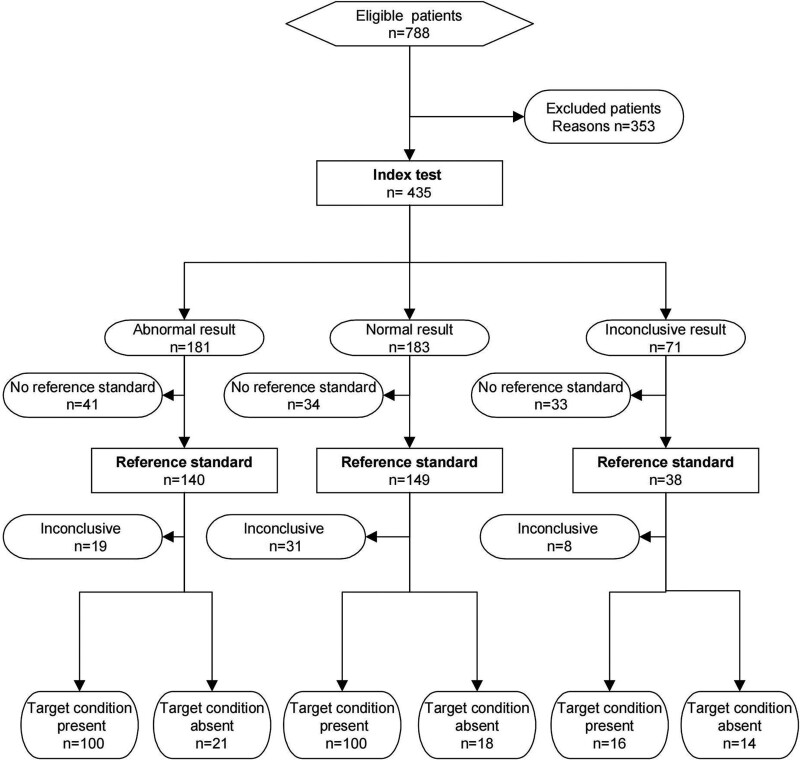
STARD flow diagram of the study.

Patients with primary VaD diagnosed by clinical and imaging examinations in the Fourth Affiliated Hospital of Jiangsu University were selected as VaD group (n = 100), and healthy subjects of similar age group (n = 100) were selected as control group. The protocol was approved by the Ethics Review Board of Jiangsu University (Clinical trial registration number: Zj202008010121). VaD and control groups excluded other diseases. There was no significant difference in the basic conditions between the 2 groups (Fig. 1 and Table 1).

**Table 1:** Characteristics and of the study population.

	VaD	Control	
n	100	100	
Gender			
Female	45	41	*P* > .05
Male	55	59	
Age, yr (SD)	67.52 (6.51)	70.34 (8.01)	*P* > .05
BMI (SD)	23.35 (3.41)	22.60 (2.55)	*P* > .05
Smoker	35	39	
Alcohol use	41	38	
VaD family history	27	21	
IL-6 (pg/mL, SD)	110.21 (12.14)	98.54 (10.07)	*P* > .05

VaD = vascular dementia.

According to Neurocognitive Index (NCI) scores, VaD patients were divided into mild group, moderate group and severe group.

According to Doppler examination, VaD patients were divided into normal IMT group and abnormal IMT group. According to D-dimer examination results, VaD patients were divided into normal D-dimer group and abnormal D-dimer group.

### 2.2. Determination method

IMT was determined by ultrasonic Doppler (ESAOTE Mylab90, Italy) and D-dimer by the Laboratory Department of the Fourth Affiliated Hospital of Jiangsu University using a fully automated biochemical analyzer (Beckman-Coulter au5800). Cognitive function was assessed by the NCI.^[6]^

### 2.3. Statistics

Statistics were calculated by SAS Visual Analytics8.4, and the measurement data were expressed by x ± s. The 2 groups were compared by *t* test, and the counting data were tested by χ^2^. The difference was statistically significant when *P* < .05.

## 3. Results

### 3.1. Differences of D-dimer and IMT among control group, mild, moderate and severe group.

**Figure 2.**
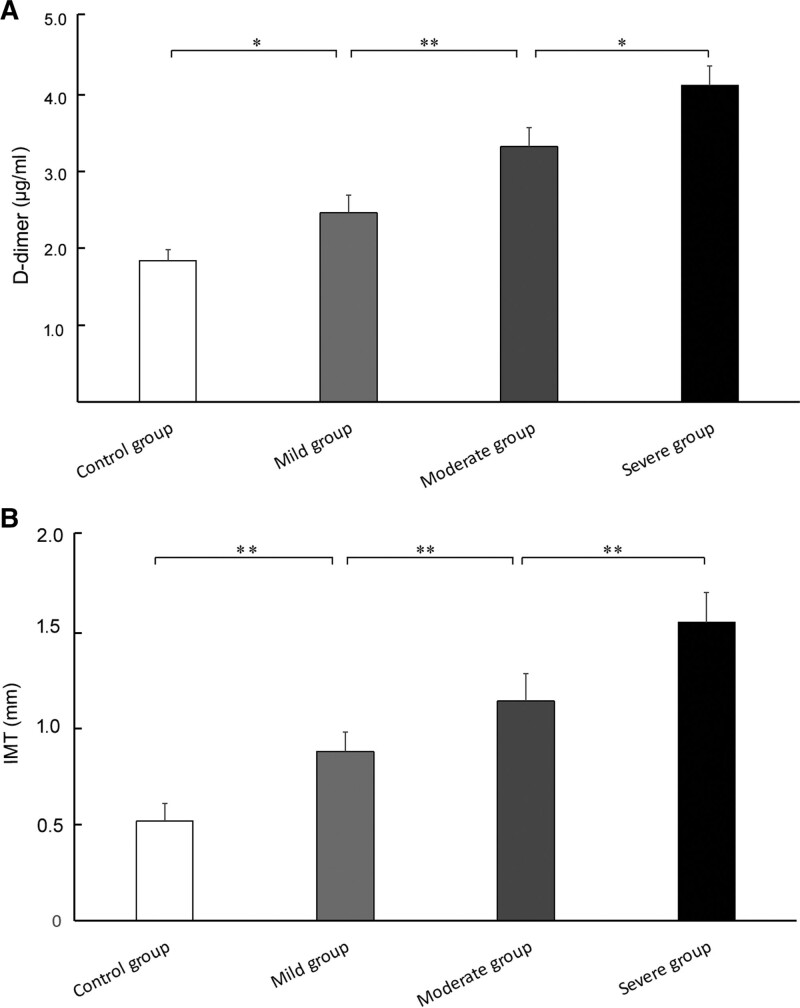
Comparison of D-dimer (A) and IMT (B) levels among control group, mild, moderate and severe group. *P < .05, **P < .01. IMT = intima-media thickness.

As shown in Figure 2A, differences of D-dimer and IMT among control group, mild, moderate and severe group were measured by the same instrument with the same parameters, have statistical significant. IMT levels in the mild were higher than the control group (*P* < .05). IMT levels in the moderate were higher than the mild group (*P* < .01). IMT levels in the severe were higher than the moderate group (*P* < .05). As shown in Figure 2B, D-dimer levels between different groups showed difference (*P* < .01).

### 3.2. Correlation between VaD/cognitive assessment scores and IMT/D-dimer levels

#### 3.2.1. Correlation between VaD and IMT/D-dimer levels.

Correlation between VaD and IMT/D-dimer levels are shown in Tables 2 and 3, respectively. The results shows D-dimer in VaD group are associated with disease, χ^2^ = 25.12, *P* < .01. The results also shows IMT in VaD group are associated with disease, χ^2^ = 83.74, *P* < .01.

**Table 2:** Correlation between D-dimer and vascular dementia.

	Condition	
	Control	VaD
D-dimer		
Normal	77	45
High	23	55

VaD = vascular dementia.

χ^2^ = 25.12, *P* < . 01.

**Table 3:** Correlation between IMT and vascular dementia.

	Condition	
	Control	VaD
IMT		
Normal	73	11
High	27	89

IMT = intima-media thickness, VaD = vascular dementia.

χ^2^ = 83.74, *P* < .01.

#### 3.2.2. Correlations between NCI and D-dimer/IMT levels in VaD are shown in Figure 3.

**Figure 3.**
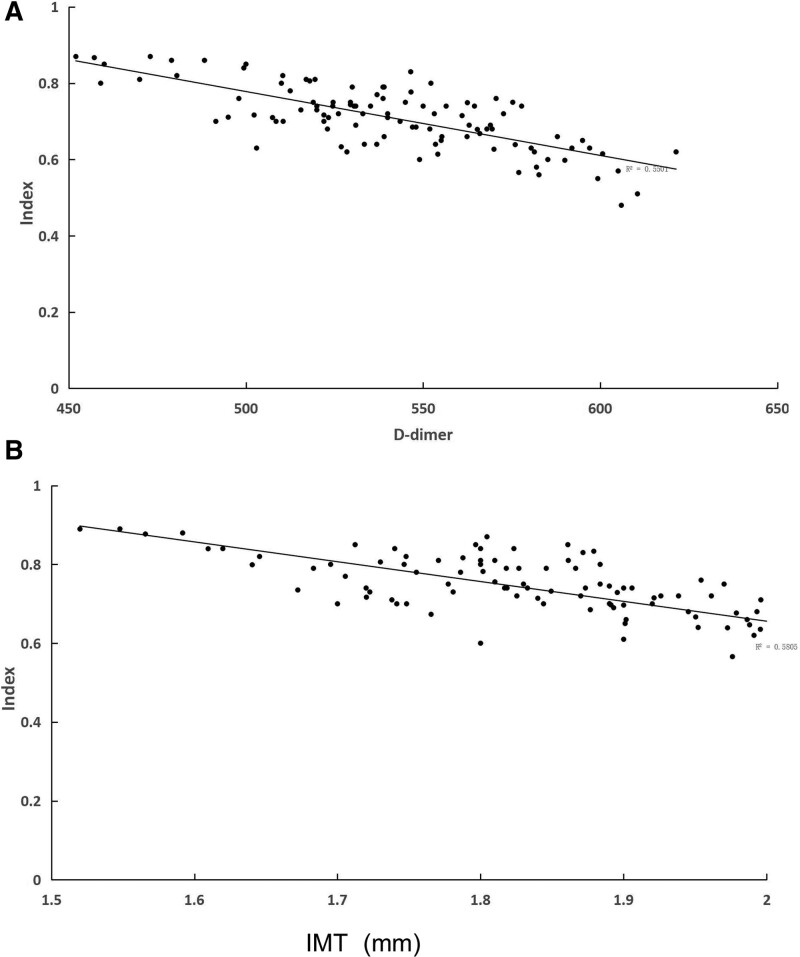
Correlation between cognitive assessment scores and D-dimer (A) and IMT (B) levels in subjects (r = −76.19, r = −74.16, *P* < .01, *P* < .01, respectively). IMT = intima-media thickness.

D-dimer and IMT levels were negatively correlated with the NCI (r = −76.19, r = −74.16, *P* < .01, *P* < .01, respectively) indicated by Pearson correlation analysis. Therefore, they were all positively correlated with the severity of the disease as shown in Figure 3.

### 3.3. Receiver operator characteristic curve of IMT and D-dimer levels for the diagnosis of patients

Analysis of the Diagnostic Value of IMT levels in Patients Receiver operator characteristic curve analysis showed that the area under the curve (AUC) of IMT levels in the diagnosis of VaD was 0.785 (95% CI: 0.746–0.885). When the best cutoff value was 1.69 mg/L and the Youden index was 0.456, the sensitivity was 88.2% and the specificity was 67.1%.

The AUC of D-dimer in the diagnosis of patients with VaD was 0.772 (95% CI: 0.678–0.866). When the best cutoff value was 92.58 g/L and the Youden index was 0.503, the sensitivity was 69.7% and the specificity was 80.6%.

**Figure 4.**
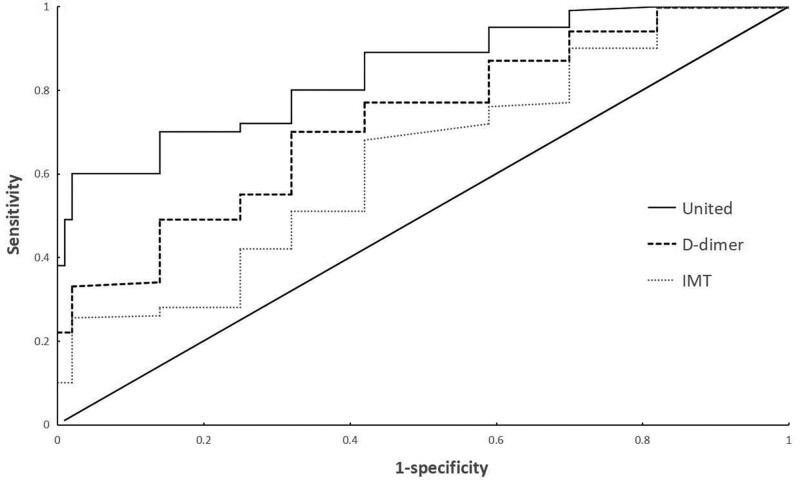
Receiver operator characteristic (ROC) curve of D-dimer and/or IMT for the diagnosis of patients. IMT = intima-media thickness.

The AUC of the 2 combined diagnoses of patients with VaD was 0.872 (95%CI: 0.801–0.921). When the Youden index was 0.580, the sensitivity was 90.2% and the specificity was 87.9% (Fig. 4).

## 4. Discussion

indicator for early diagnosis, D-dimer is not only associated with stroke and other vascular diseases but also with dementia, even is a risk factor for VaD. Furthermore, blocking coagulation factors can be useful in the management and prevention of VaD.^[4,7]^ D-dimer was associated with cognitive decline. It is interesting that many researches showed coincident results that D-dimer has no association between the risk of Alzheimer disease (AD).

Masley^[8]^ found that detection of IMT can predict the occurrence of disease before the onset of lesions. Dhamoon MS^[9]^ even found that detection of IMT can predict the occurrence of arteriosclerosis in childhood and can also be used as an indicator of efficacy. Carotid IMT is closely related to the presence and severity of intra-arterial plaque. IMT thickness was correlated with plaque severity positively. In the prognosis of IMT and early atherosclerosis for cerebral vascular diseases, age and immune system are recently considered to be the important factors in the obese patients with nonalcoholic fatty liver disease, which proved the main roles of the aging and immune system in the mechanism of cause, process and worsening of atherosclerosis.^[10]^

NCI scores display association with lower carotid IMT scores. Increased CCA IMT (4th vs 1st quartiles) was also associated with lower cognitive performance, with cognitive impairment and more pronounced subclinical atherosclerosis.

The positions of the IMT measurement are very important, in the Framingham study, greater IMT in the internal carotid artery was associated with impaired cognitive function. However, no association with the common carotid IMT and cognitive impairment.

Gustavsson AM^[5]^ found that IMT associates with VaD and other disease but not with atherosclerosis and subsequent AD dementia or AD pathology.

Cho^[2]^ alterations in IMT and plaque number are associated with an increased risk of cognitive decline as well as conversion from MCI to dementia and that blood factor analysis may aid to detect the severity of cognitive decline.

Although IMT can also predict and evaluate arteriosclerosis, the clinical significance of IMT is still controversial.^[5,11,12]^ Some scholars have proposed that in order to accurately predict and evaluate the degree of arteriosclerosis, it is necessary to detect multiple indicators at the same time. In this paper, the feasibility of the combined detection of D-dimer and IMT is determined by clinical data. The results prove that the above proposal is correct and feasible. Both D-dimer and IMT have limitations. The combined detection can increase the sensitivity of the detection. It can be used as a screening and routine check item, even evaluate the severity of cognitive decline.^[13]^

## 5. Limitation of this article

One limitation of this article is the relative less of cases and controls, which was approved by the ethnics approval in our university. Another limitation is the homogeneity of the cases, the control group was from volunteers.

## Acknowledgments

We wish to acknowledge Dr Jinjun Qian and Dr Weifang Zhang (Department of Neurology, 4th Hospital Affiliated to Jiangsu University) for his direction of clinical and laboratory analysis.

## Author contributions

**Conceptualization:** Biao Jin, Kangren Zhao.

**Data curation:** Rui Ma.

**Formal analysis:** Biao Jin, Rui Ma.

**Investigation:** Cen Su, Kangren Zhao.

**Methodology:** Haiping Xia, Kangren Zhao.

**Resources:** Cen Su.

**Software:** Biao Jin, Cen Su.

**Writing – original draft:** Haiping Xia.

**Writing – review & editing:** Rui Ma.
